# BRCAness, DNA gaps, and gain and loss of PARP inhibitor–induced synthetic lethality

**DOI:** 10.1172/JCI181062

**Published:** 2024-07-15

**Authors:** Xin Li, Lee Zou

**Affiliations:** Department of Pharmacology and Cancer Biology, Duke University School of Medicine, Durham, North Carolina, USA.

## Abstract

Mutations in the tumor-suppressor genes *BRCA1* and *BRCA2* resulting in BRCA1/2 deficiency are frequently identified in breast, ovarian, prostate, pancreatic, and other cancers. Poly(ADP-ribose) polymerase (PARP) inhibitors (PARPis) selectively kill BRCA1/2-deficient cancer cells by inducing synthetic lethality, providing an effective biomarker-guided strategy for targeted cancer therapy. However, a substantial fraction of cancer patients carrying *BRCA1/2* mutations do not respond to PARPis, and most patients develop resistance to PARPis over time, highlighting a major obstacle to PARPi therapy in the clinic. Recent studies have revealed that changes of specific functional defects of BRCA1/2-deficient cells, particularly their defects in suppressing and protecting single-stranded DNA gaps, contribute to the gain or loss of PARPi-induced synthetic lethality. These findings not only shed light on the mechanism of action of PARPis, but also lead to revised models that explain how PARPis selectively kill BRCA-deficient cancer cells. Furthermore, new mechanistic principles of PARPi sensitivity and resistance have emerged from these studies, generating potentially useful guidelines for predicting the PARPi response and design therapies for overcoming PARPi resistance. In this Review, we will discuss these recent studies and put them in context with the classic views of PARPi-induced synthetic lethality, aiming to stimulate the development of new therapeutic strategies to overcome PARPi resistance and improve PARPi therapy.

## The concepts of synthetic lethality and BRCAness

The concept of synthetic lethality was first described by the fly geneticist Calvin Bridges in 1922 (1, 2) as the combination of two genetic events that results in cell death or death of an organism. The term “synthetic lethality” is commonly used to describe a specific type of genetic interaction whereby simultaneous loss of two nonessential functions is incompatible with cell or organismal viability. Since its conception, synthetic lethality has been widely used by genetic studies in different model organisms to help with understanding the functional relationships between different genes and pathways. During the last two decades, the concept of synthetic lethality has been successfully applied to cancer therapy, allowing us to exploit various vulnerabilities of cancer cells (3). The basic rationale for this approach is that the oncogenic events in cancer cells give rise to distinct vulnerabilities, rendering cancer cells more dependent on certain genes and pathways for survival than normal cells. When the functions of these genes and pathways are disrupted, selective or preferential killing of cancer cells can be achieved. Thus, the synthetic lethality of cancer cells in a therapeutic setting is not an effect of two incompatible genetic events, but a result of a targeted drug exploiting a liability in a specific oncogenic context. A classic example of the use of synthetic lethality in cancer therapy is the development of poly(ADP-ribose) polymerase (PARP) inhibitors for the treatment of cancer patients carrying mutations in *BRCA1/2* tumor-suppressor genes (3). As we will discuss below, the loss of BRCA1/2 functions in cancer cells creates a dependency upon proper PARP functions and cycling for cell survival.

*BRCA1* and *BRCA2* are two tumor-suppressor genes frequently mutated in breast, ovarian, prostate, pancreatic, and other cancers. Familial mutations in *BRCA1/2* genes significantly increase the lifetime risk of breast (up to 85%) and ovarian (15%–56%) cancers in carriers (4). The best-known functions of BRCA1 and BRCA2 proteins are in homologous recombination (HR), a DNA repair pathway that accurately repairs DNA double-stranded breaks (DSBs) (5, 6). Loss of BRCA1/2 in cancer cells results in HR deficiency (HRD) and increased levels of DSBs in the genome, which likely fuels tumorigenesis (7). Mutations in other HR genes, such as *PALB2, RAD51C,* *RBBP8* (also known as *CtIP*), and *BRIP1* (also known as *FANCJ/BACH1*), are also found in cancers (8–11). More recent studies have revealed additional roles of BRCA1/2 in genome protection, including their functions in protecting stalled DNA replication forks (12, 13), preventing accumulation of DNA gaps (14–16), and suppressing R-loops, a 3-stranded nucleotide structure formed by stable RNA-DNA hybrids (17–19). Defects in these BRCA1/2 functions may also contribute to genomic instability in cancer cells. In addition to loss-of-function mutations in *BRCA1/2* and other HR genes, expression of these genes can also be lost in cancers through DNA hypermethylation and other mechanisms (20). Loss of BRCA1/2 and related functions in cancer is often described as “BRCAness,” which refers to both the functional defects in cancer cells and the consequent vulnerabilities (21). Levels of HRD in tumors can be scored by measuring loss of heterozygosity, telomeric allelic imbalance, and large-scale transitions, providing a biomarker for BRCAness (22). More recent approaches that combine chromosomal rearrangement signatures and base substitution signatures associated with BRCA1/2 or HR loss may provide a more effective way to predict BRCAness in tumors (23, 24). The concepts of synthetic lethality and BRCAness have established a framework for targeting *BRCA1/2* mutant tumors and other HR-deficient tumors in cancer therapy.

## Development and advancement of PARP inhibitors

The genomic instability associated with BRCAness offers an opportunity to selectively induce synthetic lethality in cancer cells (25). PARP1 was the first member of the family of PARP enzymes shown to function in DNA repair (26). By recognizing DNA nicks and ends and synthesizing poly(ADP-ribose) (PAR) chains at sites of DNA damage, PARP1 plays an important role in base excision repair (BER) and the repair of single-stranded breaks (SSBs) (27). The first PARP inhibitor (PARPi)was developed in 1975 and found to enhance the cytotoxicity of the DNA-damaging agent dimethyl sulfate (28). This observation motivated the subsequent development of additional PARPis to augment the effectiveness of conventional radiotherapy and chemotherapy. In 2005, two landmark studies by the Ashworth and Helleday labs demonstrated that BRCA1/2-deficient cancer cells are highly sensitive to PARP inhibition (29, 30). Four years later, a clinical trial demonstrated the antitumor activity of the PARPi olaparib (AZD2281) in *BRCA* mutation carriers (31). The success of these early studies inspired the development of additional PARPis by various pharmaceutical companies and clinical trials in different patient cohorts.

To date, at least eight different PARPis have been or are being tested in clinical trials (Table 1). These PARPis include olaparib (KuDOS/AstraZeneca) (31), veliparib (Abbvie) (32), rucaparib (Pfizer/Clovis) (33), niraparib (Merck/Tesaro) (34), talazoparib (Lead/Biomarin/Medivation/Pfizer) (35), pamiparib (BeiGene) (36), and venadaparib (Idience) (37). All PARPis listed inhibit both PARP1 and PARP2 proteins (38, 39), but they exhibit different potency, selectivity, and efficacy in patients. In addition, a PARP1-selective PARPi (AZD5305) developed by AstraZeneca (40) is being tested in clinical trials.

Olaparib (Lynparza; AstraZeneca) was the first clinically applied PARPi, receiving approval from the USA FDA and European Medicines Agency (EMA) for advanced ovarian cancer patients with deleterious or suspected deleterious germline *BRCA* (*gBRCA*) mutations in 2014 (41, 42). Olaparib was subsequently approved by the FDA for *gBRCA*-mutated HER2-negative metastatic breast cancer in 2018 (ClinicalTrials.gov NCT01844986), *gBRCA*-mutated pancreatic cancer in 2019 (NCT02184195), and adjuvant treatment of high-risk early breast cancer in 2022 (NCT02032823). The latest follow-up studies in the SOLO1 ovarian cancer maintenance trial showed that olaparib significantly improved the overall survival (OS) of advanced ovarian cancer patients carrying *BRCA1/2* mutations after seven years, marking it the first PARPi shown to provide an OS benefit (43). Olaparib also extended median progression-free survival (PFS) in HER2-negative breast cancer patients carrying *gBRCA* mutations in the OlympiAD trial, but the OS benefit was less pronounced than that in the ovarian cancer trial (44).

Rucaparib was approved by the FDA for the maintenance of advanced ovarian cancer patients with deleterious *BRCA* mutations. It is currently being tested in patients with metastatic castration-resistant prostate cancer with deleterious *BRCA* mutations (NCT02952534) and multiple recurrent cancer maintenance therapies (NCT01968213). A clinical trial of niraparib resulted in significantly longer PFS in ovarian cancer patients (NCT02655016), leading to the FDA approval of using niraparib in first-line maintenance therapy, regardless of HRD status (45). Talazoparib (BMN 673/Talzenna; Pfizer) was approved by the FDA for advanced breast cancer patients with *gBRCA* mutations (NCT01945775) and patients with metastatic castration-resistant prostate cancer with mutations in HR genes (NCT03395197). Veliparib is generally considered to be a weak PARPi and is primarily used in combination with platinum-based chemotherapy or radiation therapy (46). Pamiparib displayed antitumor activity in advanced ovarian cancer patients with *gBRCA* mutations (36). A trial of AZD5305 is being conducted in patients with advanced solid malignancies (NCT04644068). AZD5305 is 500-fold more selective for PARP1 than PARP2 (47). In preclinical models, AZD5305 selectively killed BRCA2-deficient cells and displayed much less hematologic toxicity than olaparib (40). The minimal side effects of AZD5305 compared with PARP1/2 dual inhibitors are likely due to the loss of PARP2 trapping–associated toxicity (see below). In the ongoing trial, AZD5305 exhibited a promising safety profile and compelling clinical activity (48). In addition to the monotherapy trials using various PARPis, several PARPis are also being tested in combination with radiotherapy, chemotherapy, and immunotherapy (recently reviewed in ref. 49). These trials may offer new opportunities to broaden the utility of PARPis and enhance their efficacy in cancer therapy.

## Mechanisms of action

### Classic models.

**Figure 1:**
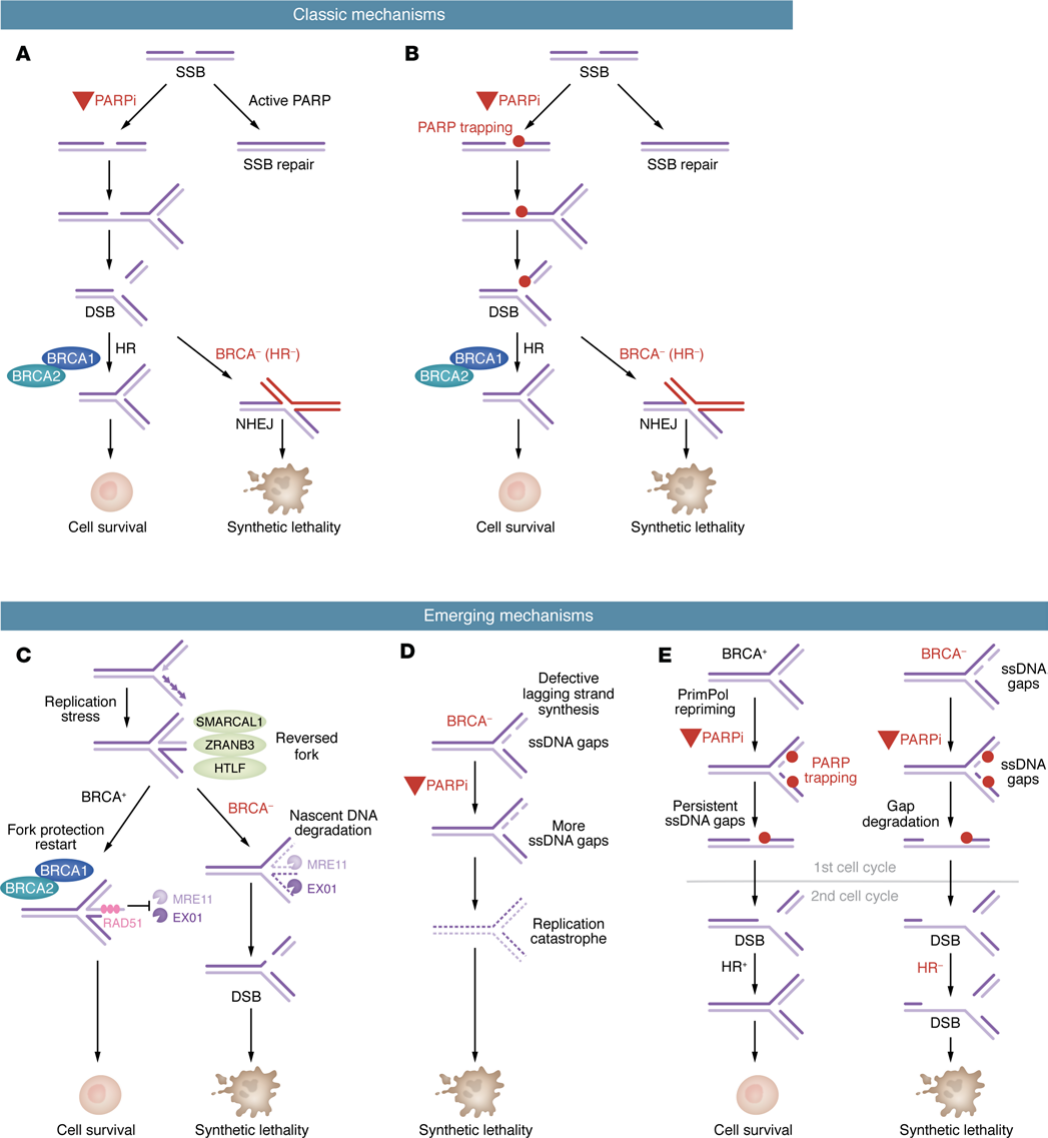
Models of PARPi-induced synthetic lethality: classic and emerging mechanisms. (**A**) A model showing how increasing SSBs in HR-defective cells leads to error-prone NHEJ, which results in cell death. (**B**) A model showing how trapping of PARP by PARPis on DNA interferes with replication. (**C**) A model showing how nascent DNA is degraded from reversed replication forks in BRCA-deficient cells. (**D**) A model showing how PARPis induce ssDNA gaps and trigger replication catastrophe in BRCA-deficient cells. (**E**) A model showing how PARPis induce persistent ssDNA gaps and collisions between gaps and replication forks in the trans-cell-cycle manner. BRCA-deficient cells fail to repair fork collapsed at gaps due to their HR defects.

Several models have been proposed to explain why PARP inhibition selectively induces synthetic lethality in BRCA1/2- or HR-deficient cancer cells (Figure 1A). The first model focused on the SSB repair function of PARP1 (29, 30). In this model, inhibition of PARP activity results in accumulation of unrepaired SSBs in the genome, giving rise to high levels of DSBs when SSBs and replication forks collide (Figure 1A). Because BRCA1/2-deficient cancer cells are HR defective, they are forced to use error-prone repair pathways (e.g., nonhomologous end joining [NHEJ]) to repair DSBs at collapsed replication forks, leading to toxic repair products and selective killing by PARPi. Thus, loss of HR and SSB repair results in synthetic lethality. Consistent with this model, an SSB repair–defective *XRCC1* mutant cell line displays high levels of spontaneous DSBs and RAD51 foci, indicating that HR is used to repair SSB-derived DSBs in HR-proficient cells (29). Furthermore, BRCA1/2-deficient cells, but not BRCA1/2-proficient cells, display higher levels of DSBs and chromosomal aberrations upon PARPi treatment, showing the ability of PARPi to selectively induce DSBs and toxic repair products in BRCA1/2-deficient cells.

A second model to explain the effects of PARPi in BRCA1/2-deficient cells was proposed following the observation that PARPis trap PARP1/2 on chromatin (38). Notably, the potency of various clinical PARPis in trapping PARP1/2 correlates with their efficacy in killing BRCA1/2-deficient cancer cells, suggesting that PARPi induces DNA damage by trapping PARP1/2 on DNA (Figure 1B). In this model, PARPi-mediated trapping of PARP1/2 at SSBs creates barriers for replication forks, increasing DSBs in both BRCA1/2-deficient and -proficient cells. BRCA1/2-deficient cells are selectively killed by PARPi because they cannot repair DSBs properly and efficiently, supporting a synthetic lethal relationship between HR loss and PARP trapping (Figure 1B). Indeed, *PARP1* mutations in BRCA1/2-deficient cells were shown to confer PARPi resistance (39), suggesting that PARP1 is the key target for trapping to kill BRCA1/2-deficient cells. Nonetheless, a recent study challenged the concept that PARP trapping by PARPi drives the killing of BRCA1/2-deficient cells (50). This study suggests that PARP functions with TIMELESS and TIPIN to alleviate transcription-replication conflicts, and the loss of this PARP function results in synthetic lethality of BRCA1/2-deficient cells.

### Emerging models.

While the roles of BRCA1/2 in HR are most extensively studied, their functions at replication forks are increasingly appreciated. Both BRCA1 and BRCA2 are important for preventing the nucleolytic degradation of nascent DNA at stalled replication forks (12, 13). The degradation of nascent DNA in BRCA-deficient cells involves MRE11 and EXO1 nucleases and requires SMARCAL1, ZRANB3, and HLTF, which convert stalled replication forks into 4-way structures known as reversed forks (51–54), suggesting that BRCA1/2 protect reversed forks from degradation by MRE11 and EXO1. Notably, when BRCA1-deficient cells acquire resistance to PARPi, they regain fork protection (55). In BRCA2-deficient cells, loss of the MLL3/4 complex protein PTIP allows cells to regain fork protection and confer PARPi resistance (56). These results suggest a model in which increased degradation of nascent DNA at reversed forks in BRCA-deficient cells exacerbates fork collapse and impairs proper fork recovery, driving genomic instability and cell death in PARP inhibition (Figure 1C).

Although BRCA1/2 protect the replication forks reversed under stress, PARP inhibition reduces the accumulation of reversed forks (57), suggesting that the defect of BRCA-deficient cells in protecting reversed forks may not be directly relevant to their sensitivity to PARPi. A number of recent studies showed that BRCA-deficient cells accumulate elevated levels of single-stranded DNA (ssDNA) or ssDNA gaps (14, 15, 58–61). Some of these studies suggested that the ssDNA gaps in BRCA-deficient cells are generated by PrimPol-mediated repriming (14, 15, 60), whereas others linked the ssDNA gaps to defective lagging strand synthesis and alterations in PCNA cycling, which interfere with chromatin assembly (16, 61). In particular, the Caldecott lab showed that PARP1 was activated by defective Okazaki fragment processing and PARP inhibition impeded lagging strand maturation during DNA replication, leading to an increase in postreplicative single-strand nicks or gaps (62). BRCA-deficient cells are also defective for the postreplicative repair of ssDNA gaps (63). Interestingly, PARP inhibition was shown to induce the accumulation of ssDNA gaps in nascent DNA in both BRCA1-proficient and -deficient cells (15). Both PrimPol-mediated repriming and defects in lagging strand maturation may contribute to the formation of PARPi-induced ssDNA gaps (15, 62). Two different models were proposed to explain how PARP inhibition may exacerbate the ssDNA gaps in BRCA-deficient cells to kill them selectively. In the first model, PARP inhibition increases ssDNA gaps in BRCA-deficient cells, which kill cells through replication catastrophe, a process triggered by extremely high levels of ssDNA (59) (Figure 1D). In the second model, PARP inhibition does not drastically increase ssDNA gaps in BRCA-deficient cells, but rather makes ssDNA gaps more persistent by trapping PARP1 (15). Of note, BRCA-deficient cells are defective for protecting ssDNA gaps from degradation by MRE11 (63). Inhibition of MRE11 in BRCA-deficient cells restores gap filling, suggesting that MRE11-mediated DNA degradation from gaps interferes with gap repair (63). In addition, PARP inhibition preferentially increases the gap-initiated nascent DNA degradation in BRCA-deficient cells (64). When ssDNA gaps with trapped PARP1 persist into mitosis and the following S phase, DSBs are formed (15, 65) (Figure 1E). BRCA-deficient cells cannot repair gap-derived DSBs efficiently and fail to activate the replication checkpoint to slow down the cell cycle; therefore, they accumulate more DSBs than BRCA-proficient cells over multiple cell cycles (15). Thus, the PARPi-induced persistent ssDNA gaps are incompatible with the HR and checkpoint defects of BRCA-deficient cells, resulting in an alternative form of synthetic lethality. This model may explain the progressive killing of BRCA-deficient cells by PARPi over time.

It should be noted that the different models above may not be mutually exclusive. Some of these models may be linked by a common underlying mechanism, and multiple mechanisms may contribute to the killing of BRCA-deficient cells by PARPi. For example, the defects of BRCA-deficient cells in gap suppression may increase the chance for PARPi to trap PARP at gaps and generate more DSBs, whereas the HR defects of BRCA-deficient cells may prevent the repair of these DSBs. This model may explain why HR defect, gap-suppression defect, and PARP trapping all contribute to the PARPi sensitivity of BRCA-deficient cells. It will be important to test and compare the above models further in future preclinical and clinical studies. The relevance of the emerging models in the PARPi resistance of BRCA-deficient cells will be discussed below.

## Potential side effects of PARPis

While PARPi-induced trapping of PARP1 may be the key driver for the killing of BRCA-deficient cells, most PARPis inhibit and trap both PARP1 and PARP2. The PARP family has 18 members (26). Among all PARP family proteins, PARP1, PARP2, and PARP3 have the most extensively studied roles in DNA repair (66). The conserved regions common to these three proteins include the DNA damage–sensing Trp-Gly-Arg–rich (WGR) region, the helical domain (HD) region, which regulates catalytic activity, and the ADP-ribosyl transferase (ART) domain. Most PARPis share a similar mode of action that leads to the selective killing of BRCA-deficient cancer cells by targeting PARP1; however, their primary targets and adverse drug reactions vary (67). The side effects of PARPis could be caused by common or unique molecular features of these compounds. Cumulating evidence suggests that PARPis also target other PARP family proteins (68, 69), potentially explaining some side effects caused by PARPis. Loss of PARP2 activity is believed to be responsible for certain hematological side effects observed in PARPi-treated patients (70). A recent study showed that talazoparib, niraparib, and rucaparib exert an allosteric effect on PARP2 that retains PARP2 at DNA breaks, suggesting that PARP2 trapping may be responsible for the side effects of these PARPis (71). Surprisingly, none of the current clinical PARPis exert the same allosteric effect on PARP1. In the future, generating PARPis capable of exerting this allosteric effect on PARP1 is likely to enhance efficacy and reduce side effects.

## Mechanisms of PARPi resistance

Despite the ability of PARPi to induce synthetic lethality in BRCA-deficient cancer cells, only half of cancer patients carrying *BRCA1/2* mutations respond to PARPi therapy (72). Furthermore, most of the patients treated with PARPi developed PARPi resistance over time. Thus, the preexisting and acquired resistance to PARPi represents a major obstacle to PARPi therapy in the clinic. In this section, we will discuss the three main classes of PARPi resistance mechanisms, with an emphasis on the recently discovered mechanisms associated with the restoration of fork protection and gap suppression.

### Mechanisms rendering PARPis ineffective.

**Figure 2:**
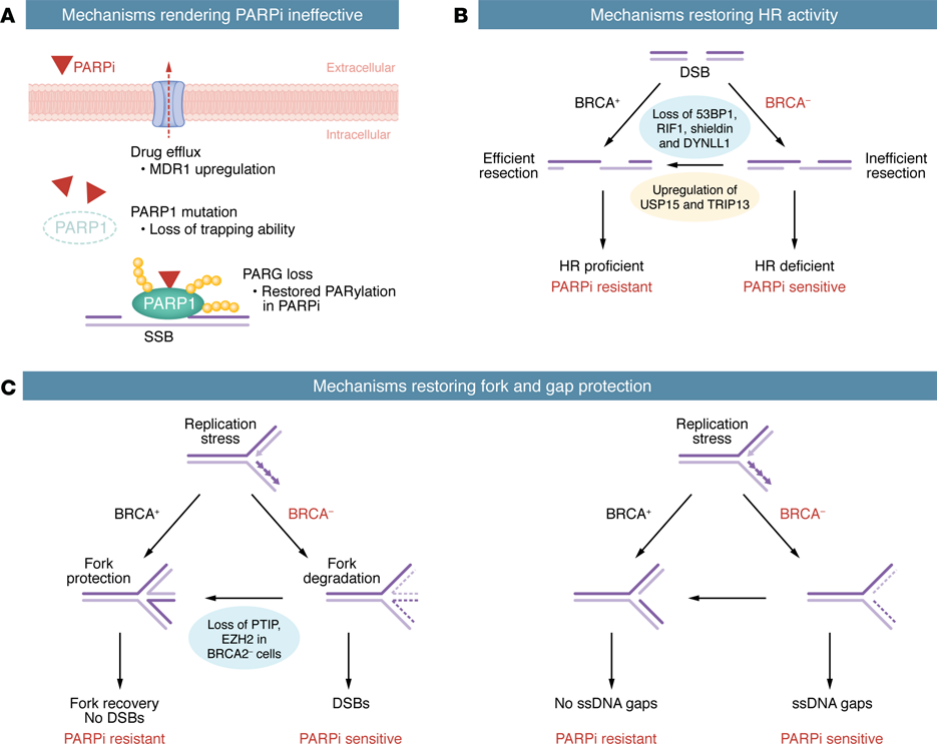
Mechanisms of PARPi resistance. An overview of the three classes of PARPi resistance mechanisms, with subcategories and examples described. (**A**) Mechanisms rendering PARPi ineffective. (**B**) Mechanisms restoring HR activity. (**C**) Mechanisms restoring fork and gap protection.

BRCA-deficient cancer cells can acquire PARPi resistance through several mechanisms that render PARPis ineffective (Figure 2A). For example, upregulation of drug efflux pumps can remove PARPi from cells and cause PARPi resistance in mouse tumor models (73). Several mutations in PARP1, the key target of PARPis, are shown to confer PARPi resistance in BRCA-deficient cell lines, possibly by altering the DNA-binding properties of PARP1 and reducing PARP1 trapping (39). PARP enzymes catalyze the addition of PAR chains onto themselves and their substrates (a process known as PARylation), whereas PAR glycohydrolase (PARG) removes PAR chains and antagonizes PARP activity. Loss of PARG protein restores PARylation in PARPi-treated cells and counters PARPi-mediated synthetic lethality (74). Interestingly, the contributions of PARG loss to PARPi resistance are different in BRCA1- and BRCA2-deficient cells (75). While PARG loss is a major mechanism for PARPi resistance in BRCA2-deficient cells, the resistance of BRCA1-deficient cells is mainly caused by restoration of HR and protection of gaps and forks (see sections below). Collectively, the resistance mechanisms above reduce the effectiveness of PARPis, but they don’t necessarily alter the DNA repair in BRCA-deficient cells.

### Mechanisms restoring HR activity.

BRCA-deficient cancer cells can also acquire PARPi resistance by restoring HR activity (Figure 2B). Secondary “revertant” mutations in *BRCA1/2* genes, which restore the open reading frames after frameshift mutations, increase HR activity and confer PARPi resistance in vitro and are found in PARPi-resistant patients (76, 77). Additionally, another prevalent resistance mechanism, observed particularly in *BRCA1* mutant sporadic triple-negative breast tumors, is *BRCA1* promoter demethylation that restores *BRCA1* expression (78). Moreover, BRCA1-deficient cells can restore HR by increasing DNA end resection through the loss of resection inhibitors, such as 53BP1 (79, 80), REV7 (81), and dynein light chain 1 protein (DYNLL1) (82), or by upregulating resection enhancers, such as the ATPase TRIP13 (83) and ubiquitin carboxyl terminal hydrolase 15 (USP15) (84). Loss of the 53BP1 interacting protein RIF1 and the shieldin complex, which comprises REV7, SHLD1, SHLD2, and SHLD3, also increases HR in BRCA1-deficient cells by increasing end resection (85).

### Mechanisms restoring fork and gap protection.

As discussed above, both BRCA1 and BRCA2 are important for protecting reversed replication forks from nucleolytic degradation. A BRCA2 point mutant in the C terminus (S3291A) is unable to stabilize RAD51 filaments and fails to protect replication forks, suggesting that BRCA2 functions through RAD51 in this process (12). Cells expressing a RAD51 mutant (T131P) defective for fork protection but proficient for HR are modestly sensitive to PARP inhibition (53, 54, 86). Interestingly, in a panel of PARPi-resistant cell lines derived from a BRCA1-deficient cell line, protection of replication forks is uniformly restored (55). In BRCA2-deficient cells, loss of PTIP restores fork protection and renders cells resistant to PARPi (56). Furthermore, loss of the histone methyltransferase EZH2 also prevents fork degradation in BRCA2-deficient cells and confers PARPi resistance (87). These results suggest that degradation of reversed replication forks in BRCA-deficient cells may be a key determinant for PARPi sensitivity (Figure 2C). Consistent with this hypothesis, in organoids derived from PARPi-resistant, HR-defective ovarian cancer patients, fork protection is restored (88). Despite the strong correlation between fork degradation and PARPi sensitivity, whether reversed forks are the structure triggering nascent DNA degradation in PARP inhibition remains unclear. Notably, the standard fork degradation assay is done in the presence of hydroxyurea (HU), a replication inhibitor, but not in the presence of PARPi (12). In addition, PARP inhibition prevents the accumulation of reversed forks by allowing the RECQ1 helicase to resolve them (57), suggesting that reversed forks are unlikely to be the structure degraded in BRCA-deficient cells. A recent study showed that depletion of RADX, an antagonizer of RAD51, partially suppressed ssDNA formation in cells expressing the RAD51 T131P mutant, but did not alter PARPi sensitivity (59), arguing that fork degradation is not always correlated with PARPi sensitivity.

In addition to protecting reversed replication forks, BRCA1 and BRCA2 also suppress the accumulation of ssDNA gaps during replication (14, 15, 58–61). Several studies suggested that ssDNA gaps in BRCA-deficient cells are the key determinants of PARPi sensitivity, though different models were proposed to explain PARPi sensitivity in this context (14, 15, 59, 61). LIG3 loss promotes formation of MRE11-mediated postreplicative ssDNA gaps in BRCA1-deficient cells exposed to PARPi and increases PARPi sensitivity in BRCA-deficient cells and tumors (89), suggesting that the DNA degradation at gaps is important for PARPi-induced cell death. Notably, PARPi preferentially induced gap-initiated nascent DNA degradation in BRCA-deficient cells (64), suggesting that PARPi may impose synthetic lethality to BRCA1-deficient cells through gap-derived DNA damage (Figure 2C). In PARPi-resistant cell lines derived from multiple BRCA1-deficient cell lines, the suppression of ssDNA or gaps is restored in the presence of PARPi (59, 64). Because PARP inhibition induces ssDNA gaps and renders gaps persistent (15), ssDNA gaps, rather than reversed forks, are likely the relevant structure triggering nascent DNA degradation in BRCA-deficient cells after PARPi treatment. When analyzed in patient-derived cells and organoids, PARPi-induced and gap-initiated nascent DNA degradation may serve as a useful biomarker for predicting PARPi sensitivity in patients.

It is worth noting that the protection of replication forks and ssDNA gaps by BRCA1/2 may be mechanistically linked. In the absence of BRCA1/2, both reversed forks and ssDNA gaps are resected by MRE11 (12, 51, 63). It is possible that BRCA1/2 enable RAD51 loading/stabilization at reversed forks and ssDNA gaps to protect them from MRE11 (Figure 2C) (15, 54, 55, 63, 64), which would explain why fork protection in HU and gap protection in PARPi are largely correlated. A recent study showed that a BRCA2 mutant defective for HR, gap suppression, and fork protection failed to suppress genomic instability in cells and tumor formation in mice, but another BRCA2 mutant defective for only gap suppression and fork protection remained largely functional for suppressing genomic instability and tumor formation (90). These results suggest that loss of gap suppression and fork protection is insufficient for tumorigenesis, and loss of HR is an additional necessary step. Thus, the BRCA-deficient cells in tumors are likely defective for all three functions and both the defects in HR and protection of gaps and forks may contribute to PARPi sensitivity. Finally, it should be mentioned that different assays have been used to analyze the ssDNA exposure or gap formation at or behind replication forks (14, 15, 59, 63, 64). While the DNA fiber assay coupled with S1 nuclease digestion specifically analyzes ssDNA gaps, the exposure of BrdU/CldU-labeled ssDNA under nondenaturing conditions measures ssDNA exposure through multiple mechanisms. Whether ssDNA exposure is driven by gaps in various contexts needs to be examined carefully.

## Combination therapies to overcome PARPi resistance

When BRCA-deficient cells become PARPi resistant, their defects in HR, fork protection, and gap protection are often partially or fully reverted, suggesting that these restored activities may contribute to the resistance. Additionally, BRCA-deficient but PARPi-resistant cells may be increasingly dependent on alternative DSB repair pathways for survival. These possibilities have fueled the development of strategies to overcome PARPi resistance by combining PARPi with other DNA damage response (DDR) drugs.

### Combinations of PARPi with ATR/Chk1 inhibitors.

The ataxia telangiectasia and Rad3-related (ATR) kinase, a master regulator of the replication stress response, plays a crucial role in stabilizing replication forks (64, 91). In addition to inducing fork instability, ATR inhibition also blocks HR at DSBs by preventing RAD51 recruitment (92). Thus, ATR inhibitors (ATRis) may be the ideal drugs to increase replication fork collapse and simultaneously prevent the repair of resulting DSBs.

**Figure 3:**
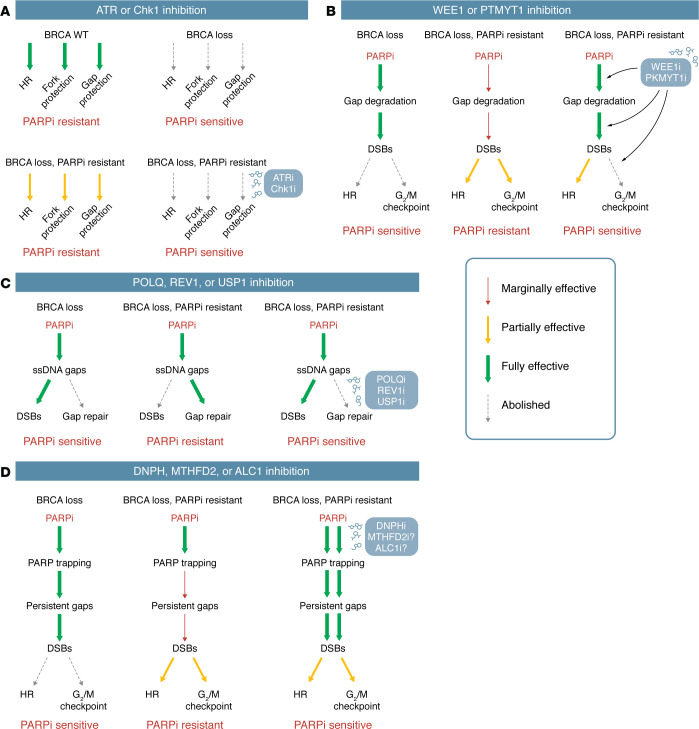
Combination therapies to overcome PARPi resistance. (**A**) ATR and Chk1 inhibitors (ATRi, Chk1i) overcome PARPi resistance by disrupting restored HR, fork-protection, and gap-protection activities. (**B**) WEE1 and PTMYT1 inhibitors (WEE1i, PKMYT1i) may overcome PARPi resistance by increasing replication and overriding the G_2_/M checkpoint. (**C**) POLQi overcomes PARPi resistance by blocking alt-EJ and/or ssDNA gap repair. REV1 and USP1 inhibitors (REV1i, USP1i) may also overcome PARPi resistance by blocking ssDNA gap repair. (**D**) Inhibition of DNPH, MTHFD2, and ALC1 (DNPHi, MTHFD2i, ALC1i) may overcome PARPi resistance by increasing PARP trapping. More studies are needed to confirm whether MTHFD2i and ALC1 can overcome resistance.

In a panel of PARPi-resistant cell lines derived from a BRCA1-deficient cell line, ATRi effectively suppressed the restored HR and fork protection activities, resensitizing the resistant cells to PARPi (55). In addition, BRCA1-deficient, PARPi-resistant cells also displayed restored abilities to prevent gap-initiated nascent DNA degradation and to activate the ATR checkpoint upon PARPi-induced fork collapse (15, 64). Again, these restored activities in the resistant cells are suppressed by ATRis. These results raise the possibility that a common underlying mechanism may be responsible for the various phenotypes associated with PARPi resistance and that this mechanism is disrupted by ATR inhibition. The exact mechanism that drives PARPi resistance in BRCA-deficient cells remains unclear, but restored RAD51 loading to ssDNA gaps and collapsed forks would alleviate PARPi-induced DNA damage. Indeed, RAD51 foci were observed in the cancer cells from *BRCA1/2* mutant patients that acquired PARPi resistance (93). Importantly, ATR inhibition abolishes the restored RAD51 foci and the loading of RAD51 to replication forks in BRCA1-deficient, PARPi-resistant cancer cells (55), supporting the idea that ATR inhibition disrupts a common RAD51-mediated mechanism driving PARPi resistance (Figure 3A).

The ability of ATR inhibition to overcome the PARPi resistance of BRCA1/2-deficient cells is seen not only in cell lines, but also in patient-derived xenografts (PDXs) and organoids of BRCA-deficient tumors (88, 94, 95). Inhibitors of Chk1, the effector kinase of ATR, have similar effects in PARPi-resistant cell lines and PDXs (Figure 3A) (94, 96). In a clinical trial, the combination of ATRi and PARPi showed efficacy in HR-deficient, PARPi-resistant, high-grade serous ovarian cancer (HGSOC) patients (97). Several ATRis are being tested in clinical trials (98), providing opportunities to overcome PARPi resistance in the near future.

### Combinations of PARPi with WEE1/PKMYT1 inhibitors.

The tyrosine kinase WEE1 inhibits both CDK1 and CDK2 by phosphorylating the tyrosine 15 of these two kinases (99). WEE1 inhibitor (WEE1i) induces hyperactivation of CDK1/2 and overrides the G_2_/M DNA damage checkpoint, leading to excessive replication origin firing, replication catastrophe in S phase, and mitotic catastrophe in mitosis (99, 100). The combination of WEE1i and PARPi overcomes PARPi resistance in breast and ovarian cancer models (101). WEE1i and PARPi also display a synergy in preclinical models of BRCA wild-type triple-negative breast cancer (TNBC) by activating antitumor immune responses (102). WEE1 inhibition also increases PARPi sensitivity in BRCA wild-type pancreatic cancer cells (103) and HGSOC cells (104), suggesting a utility of WEE1i as a PARPi sensitizer. A recent study suggested that PKMYT1, another member of the WEE kinase family regulating the G_2_/M transition, is a promising therapeutic target in cancer cells overexpressing cyclin E1, which are under high replication stress (105). PKMYT1 inhibitor (PKMYT1i) induces unscheduled activation of CDK1 in S phase, driving cells into mitosis before completion of DNA replication. It is interesting to note that PARPi-resistant, BRCA1-deficient cells regain the abilities to suppress ssDNA and activate the checkpoint response (15, 59, 64) and that the abilities of WEE1i and PTMYT1i to increase replication and override the checkpoint may enable them to revert the changes in PARPi-resistant cells and overcome PARPi resistance (Figure 3B).

### Combinations of PARPi with inhibitors of gap repair.

DNA polymerase θ (POLθ or POLQ) plays a crucial role in repairing DSBs through the alternative end-joining (alt-EJ) pathway (also known as the microhomology-mediated end-joining or MMEJ pathway). The expression of POLQ is upregulated in HR-defective epithelial ovarian cancers, suggesting that alt-EJ functions as a backup DSB-repair pathway to compensate for the loss of HR (106). POLQ is also shown to fill in ssDNA gaps in BRCA-deficient cancer cells (107). The antibiotic novobiocin (NVB) inhibits POLQ by binding to its ATPase domain and overcomes the PARPi-resistance of BRCA-deficient cells and PDXs (108). ART558, which inhibits the polymerase activity of POLQ, also overcomes the PARPi resistance of BRCA-deficient tumors (109). Notably, POLQ inhibitors (POLQis) induce high levels of ssDNA in PARPi-resistant, BRCA-deficient cells, suggesting that they prevent the repair of resected DSB ends and/or ssDNA gaps. Like POLQ, the translesion synthesis (TLS) pathway is also implicated in the repair of ssDNA gaps in BRCA1-deficient cells (14). TLS is initiated by PCNA monoubiquitylation at stalled forks or gaps, which allows the recruitment of REV1 and several TLS DNA polymerases to bypass various DNA lesions. JH-RE-06, a TLS inhibitor that disrupts the interaction between REV1 and POLζ, preferentially kills BRCA1 mutant cells and overcomes their PARPi resistance (14). Inhibition of USP1, a deubiquitylase of PCNA, leads to persistent PCNA mono- and polyubiquitylation and fork instability, preferentially killing BRCA-deficient cells (110). Recent studies showed that USP1 inhibitors (USP1is) increase ssDNA gaps in BRCA1-deficient cells, synergize with PARPi in killing BRCA-deficient cells, and overcome PARPi resistance in BRCA1-deficient cells and PDXs (111, 112). Together, these results suggest that combining PARPi with inhibitors of ssDNA gap repair is a promising strategy for overcoming the PARPi resistance of BRCA-deficient cells (Figure 3C).

### Combinations of PARPi with drugs increasing PARP trapping.

DNPH1 (2′-deoxynucleoside 5′-monophosphate *N*-glycosidase, also known as RCL) is an enzyme that eliminates the cytotoxic hydro5-hydroxymethyl-deoxyuridine (hmdU) monophosphate. Inhibition of DNPH leads to increased hmdU misincorporation, PARP trapping, and fork collapse in BRCA1-deficient cells (113). Consequently, DNPH inhibitor (DNPHi) preferentially kills BRCA1-deficient cancer cells and overcomes their PARPi resistance. Notably, the effects of DNPH1 are dependent on the SMUG glycosylase, suggesting that DNA nicks or gaps are involved. Somewhat analogously, inhibition of the folate metabolism enzyme methylenetetrahydrofolate dehydrogenase/cyclohydrolase (MTHFD2) leads to an imbalanced dUTP:dTTP pool, increased replication stress, and preferential killing of acute myeloid leukemia (AML) cells (114). It would be interesting to determine whether MTHFD2 inhibitor synergizes with PARPi in BRCA-deficient cells to overcome their PARPi resistance. The trapping of PARP by PARPi is also stimulated by the loss of PAR-binding chromatin remodeling factor ALC1 (CHD1L) (115–117), likely due to the decrease of chromatin accessibility at DNA damage sites and reduced recruitment of repair proteins. Loss of ALC1 drastically increases the PARPi sensitivity of BRCA-deficient cells and overcomes their PARPi resistance, making ALC1 an attractive therapeutic target (Figure 3D). The studies on ALC1 suggest that nucleosome remodeling, by influencing the repair of DNA nicks or gaps, is a key determinant of PARP trapping and the PARPi sensitivity of HR-deficient cells.

It is worth noting that recent CRISPR/Cas9 loss-of-function screens have served as a powerful and unbiased tool to explore synthetic lethal interactions with PARPis in BRCA-proficient and -deficient cells (118). In addition to ALC1 (115), RNase H2 was identified as a strong synthetic lethal screen hit with olaparib (119). Loss of RNase H2 renders cells hypersensitive to PARPi and also selectively kills BRCA1/2-deficient cells. CRISPR screens in prostate cancer cells revealed that loss of MMS22L drastically increases PARPi sensitivity (120). On the other hand, CRISPR screens also revealed mechanisms of PARPi resistance, such as point mutations in PARP1 (39), loss of CHK2 (120), and loss of ARH3 (121).

It is also important to note that, while combinations of PARPis with other targeted drugs may overcome PARPi resistance, these drug combinations may also increase general cellular toxicity and side effects, including hematological toxicity. Optimization of drug scheduling and dosing is likely important for achieving the maximal efficacy of these combination therapies. It remains unclear whether the selectivity of these combinations toward BRCA-deficient cells are reduced or enhanced compared with PARPis, which is an important question to address in clinical trials.

## Expanding application of PARPis beyond BRCA1/2-mutated cancers

While mutations in *BRCA1/2* and other HR genes are useful biomarkers for PARPi therapy, it has become increasingly clear that many other common oncogenic events can also alter the PARPi sensitivity of cancer cells. Some of the oncogenic events may induce BRCAness by compromising HR or increasing replication stress indirectly, whereas others may promote HR to allow cancer cells to survive genomic instability. Both types of oncogenic events may provide opportunities for PARPi therapy. For example, mutations in the *PTEN* tumor suppressor lead to defective HR and increased PARPi sensitivity (122). Oncogenic mutants of isocitrate dehydrogenase 1 and 2 (IDH1/2) induce accumulation of the oncometabolite 2-hydroxyglutarate (2-HG), which impairs HR and generates heterochromatin-dependent DNA replication stress, making IDH1/2 mutant cancer cells hypersensitive to PARPi (123, 124). Ewing’s sarcomas expressing the EWS-FLI1 or EWS-ERG fusion oncogene are also sensitive to PARP inhibition, possibly due to R-loops and HR defects (125, 126). In castration-resistant prostate cancer, inhibition of androgen receptor (AR) induces HR defects and confers PARPi sensitivity (127, 128), suggesting that AR signaling promotes cancer cell survival by enhancing HR. Given the relevance of ssDNA gaps for the PARPi sensitivity of BRCA-deficient cells, it would be important to test to determine whether the oncogenic events above alter gap levels. Of note, IDH1/2 mutants were recently shown to induce ssDNA gaps (125). Expression of APOBEC3A, a driver of mutagenesis in multiple tumor types, also induces ssDNA gaps and confers PARPi sensitivity (129).

## Conclusions and perspectives

As discussed above, recent studies have revealed that the PARPi-induced synthetic lethality in BRCA-deficient cells can be gained or lost under various conditions, shedding light on the mechanism underlying this phenomenon. While the HR defect of BRCA-deficient cancer cells is clearly relevant to their sensitivity to PARPi (130, 131), the accumulation of ssDNA gaps in BRCA-deficient cells and the trapping of PARP at gaps are also determinants of PARPi sensitivity (15, 58, 59, 90). Given that loss of the HR activity of BRCA2 is critical for tumorigenesis (90), most if not all BRCA-deficient tumors are likely defective for both HR and gap suppression. We propose that both the HR and gap-protection defects of BRCA-deficient cells contribute to their PARPi sensitivity. The gap-protection defect of BRCA-deficient cells increases PARP trapping and DSB formation, whereas the HR defect prevents the repair of these DSBs. When gap suppression is compromised but HR remains largely proficient (e.g., in BRCA2 S3219A and RAD51 T131P mutant cells), PARPi-induced synthetic lethality is profoundly weaker than that in double-defective cells. Thus, PARPi-induced synthetic lethality is not a switch of cell death, but a quantitative dial of cell fitness. This model helps explain why quantitative changes in ssDNA gaps and PARP trapping may affect the PARPi sensitivity of BRCA-deficient cells. According to this model, reductions in ssDNA gaps and PARP trapping or increases in DSB repair through HR or alt-EJ are the main mechanisms dialing up PARPi resistance. On the other hand, strategies to increase ssDNA gaps and PARP trapping or inhibit HR and alt-EJ would dial down PARPi resistance. In addition, the levels of ssDNA gaps in cancer cells may quantitatively reflect the replication stress that drives PARPi sensitivity. It should be noted that the effects of PARPi on BRCA-deficient tumors in vivo also involve antitumor immunity (132, 133). This finding raises the possibility that the immunosuppression in the tumor microenvironments may contribute to PARPi resistance (134), whereas enhancing antitumor immunity may help overcome PARPi resistance. Future studies testing the models and possibilities above in preclinical and clinical settings would likely improve the efficacy of PARPi therapy in the clinic.

## Figures and Tables

**Table 1:**
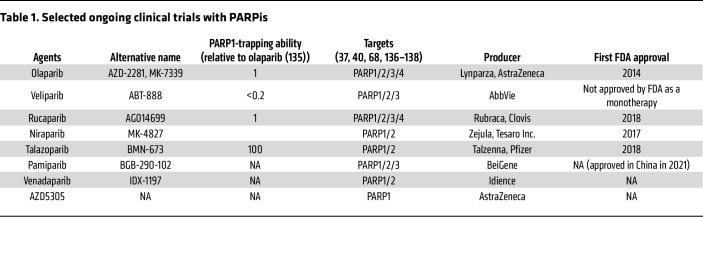
Selected ongoing clinical trials with PARPis

## Version 1 07/15/2024

Electronic publication

## References

[1] Nijman SM (2011). Synthetic lethality: general principles, utility and detection using genetic screens in human cells. FEBS Lett.

[2] Bridges CB (1922). The origin of variations in sexual and sex-limited characters. Am Nat.

[3] Lord CJ, Ashworth A (2017). PARP inhibitors: synthetic lethality in the clinic. Science.

[4] King M-C (2003). Breast and ovarian cancer risks due to inherited mutations in BRCA1 and BRCA2. Science.

[5] Prakash R (2015). Homologous recombination and human health: the roles of BRCA1, BRCA2, and associated proteins. Cold Spring Harb Perspect Biol.

[6] Venkitaraman AR (2002). Cancer susceptibility and the functions of BRCA1 and BRCA2. Cell.

[7] Nielsen FC (2016). Hereditary breast and ovarian cancer: new genes in confined pathways. Nat Rev Cancer.

[8] Cantor SB, Guillemette S (2011). Hereditary breast cancer and the BRCA1-associated FANCJ/BACH1/BRIP1. Future Oncol.

[9] Meindl A (2010). Germline mutations in breast and ovarian cancer pedigrees establish RAD51C as a human cancer susceptibility gene. Nat Genet.

[10] Antoniou AC (2014). Breast-cancer risk in families with mutations in PALB2. N Engl J Med.

[11] Zarrizi R (2020). Germline RBBP8 variants associated with early-onset breast cancer compromise replication fork stability. J Clin Invest.

[12] Schlacher K (2011). Double-strand break repair-independent role for BRCA2 in blocking stalled replication fork degradation by MRE11. Cell.

[13] Schlacher K (2012). A distinct replication fork protection pathway connects Fanconi anemia tumor suppressors to RAD51-BRCA1/2. Cancer Cell.

[14] Taglialatela A (2021). REV1-Polζ maintains the viability of homologous recombination-deficient cancer cells through mutagenic repair of PRIMPOL-dependent ssDNA gaps. Mol Cell.

[15] Simoneau A (2021). The *trans* cell cycle effects of PARP inhibitors underlie their selectivity toward BRCA1/2-deficient cells. Genes Dev.

[16] Cong K, Cantor SB (2022). Exploiting replication gaps for cancer therapy. Mol Cell.

[17] Hatchi E (2015). BRCA1 recruitment to transcriptional pause sites is required for R-loop-driven DNA damage repair. Mol Cell.

[18] Bhatia V (2014). BRCA2 prevents R-loop accumulation and associates with TREX-2 mRNA export factor PCID2. Nature.

[19] Shivji MK (2018). BRCA2 regulates transcription elongation by RNA polymerase II to prevent R-loop accumulation. Cell Rep.

[20] Swisher EM (2009). Methylation and protein expression of DNA repair genes: association with chemotherapy exposure and survival in sporadic ovarian and peritoneal carcinomas. Mol Cancer.

[21] Turner N (2004). Hallmarks of ‘BRCAness’ in sporadic cancers. Nat Rev Cancer.

[22] Telli ML (2016). Homologous recombination deficiency (HRD) score predicts response to platinum-containing neoadjuvant chemotherapy in patients with triple-negative breast cancer. Clin Cancer Res.

[23] Davies JO (2017). How best to identify chromosomal interactions: a comparison of approaches. Nat Methods.

[24] Polak P (2017). A mutational signature reveals alterations underlying deficient homologous recombination repair in breast cancer. Nat Genet.

[25] Jackson SP, Bartek J (2009). The DNA-damage response in human biology and disease. Nature.

[26] Lüscher B (2022). ADP-ribosyltransferases, an update on function and nomenclature. FEBS J.

[27] Ray Chaudhuri A, Nussenzweig A (2017). The multifaceted roles of PARP1 in DNA repair and chromatin remodelling. Nat Rev Mol Cell Biol.

[28] Purnell MR, Whish W (1980). Novel inhibitors of poly(ADP-ribose) synthetase. Biochem J.

[29] Bryant HE (2005). Specific killing of BRCA2-deficient tumours with inhibitors of poly(ADP-ribose) polymerase. Nature.

[30] Farmer H (2005). Targeting the DNA repair defect in BRCA mutant cells as a therapeutic strategy. Nature.

[31] Fong PC (2009). Inhibition of poly(ADP-ribose) polymerase in tumors from BRCA mutation carriers. N Engl J Med.

[32] Coleman RL (2019). Veliparib with first-line chemotherapy and as maintenance therapy in ovarian cancer. N Engl J Med.

[33] Coleman RL (2017). Rucaparib maintenance treatment for recurrent ovarian carcinoma after response to platinum therapy (ARIEL3): a randomised, double-blind, placebo-controlled, phase 3 trial. Lancet.

[34] González-Martín A (2019). Niraparib in patients with newly diagnosed advanced ovarian cancer. N Engl J Med.

[35] Litton JK (2018). Talazoparib in patients with advanced breast cancer and a germline BRCA mutation. N Engl J Med.

[36] Wu X (2022). Pamiparib monotherapy for patients with germline BRCA1/2-mutated ovarian cancer previously treated with at least two lines of chemotherapy: a multicenter, open-label, phase II study. Clin Cancer Res.

[37] Lee M (2023). Venadaparib is a novel and selective PARP inhibitor with improved physicochemical properties, efficacy, and safety. Mol Cancer Ther.

[38] Murai J (2012). Trapping of PARP1 and PARP2 by clinical PARP inhibitors. Cancer Res.

[39] Pettitt SJ (2018). Genome-wide and high-density CRISPR-Cas9 screens identify point mutations in PARP1 causing PARP inhibitor resistance. Nat Commun.

[40] Illuzzi G (2022). Preclinical characterization of AZD5305, a next-generation, highly selective PARP1 inhibitor and trapper. Clin Cancer Res.

[41] Ledermann J (2014). Olaparib maintenance therapy in patients with platinum-sensitive relapsed serous ovarian cancer: a preplanned retrospective analysis of outcomes by BRCA status in a randomised phase 2 trial. Lancet Oncol.

[42] Kaufman B (2015). Olaparib monotherapy in patients with advanced cancer and a germline BRCA1/2 mutation. J Clin Oncol.

[43] DiSilvestro P (2023). Overall survival with maintenance olaparib at a 7-year follow-up in patients with newly diagnosed advanced ovarian cancer and a BRCA mutation: The SOLO1/GOG 3004 Trial. J Clin Oncol.

[44] Robson M (2017). Olaparib for metastatic breast cancer in patients with a germline BRCA mutation. N Engl J Med.

[45] Mirza MR (2016). Niraparib maintenance therapy in platinum-sensitive, recurrent ovarian cancer. N Engl J Med.

[46] Pommier Y (2016). Laying a trap to kill cancer cells: PARP inhibitors and their mechanisms of action. Sci Transl Med.

[47] Johannes JW (2021). Discovery of 5-{4-[(7-Ethyl-6-oxo-5,6-dihydro-1,5-naphthyridin-3-yl)methyl]piperazin-1-yl}-*N*-methylpyridine-2-carboxamide (AZD5305): A PARP1-DNA trapper with high selectivity for PARP1 over PARP2 and other PARPs. J Med Chem.

[48] Yap TA (2022). Prevalence of homologous recombination deficiency among patients with germline RAD51C/D breast or ovarian cancer. JAMA Netw Open.

[49] Bhamidipati D (2023). PARP inhibitors: enhancing efficacy through rational combinations. Br J Cancer.

[50] Petropoulos M (2024). Transcription-replication conflicts underlie sensitivity to PARP inhibitors. Nature.

[51] Lemaçon D (2017). MRE11 and EXO1 nucleases degrade reversed forks and elicit MUS81-dependent fork rescue in BRCA2-deficient cells. Nat Commun.

[52] Taglialatela A (2017). Restoration of replication fork stability in BRCA1- and BRCA2-deficient cells by inactivation of SNF2-family fork remodelers. Mol Cell.

[53] Mijic S (2017). Replication fork reversal triggers fork degradation in BRCA2-defective cells. Nat Commun.

[54] Kolinjivadi AM (2017). Smarcal1-mediated fork reversal triggers Mre11-dependent degradation of nascent DNA in the absence of Brca2 and stable Rad51 nucleofilaments. Mol Cell.

[55] Yazinski SA (2017). ATR inhibition disrupts rewired homologous recombination and fork protection pathways in PARP inhibitor-resistant BRCA-deficient cancer cells. Genes Dev.

[56] Ray Chaudhuri A (2016). Replication fork stability confers chemoresistance in BRCA-deficient cells. Nature.

[57] Berti M (2013). Human RECQ1 promotes restart of replication forks reversed by DNA topoisomerase I inhibition. Nat Struct Mol Biol.

[58] Panzarino NJ (2021). Replication gaps underlie BRCA deficiency and therapy response. Cancer Res.

[59] Cong K (2021). Replication gaps are a key determinant of PARP inhibitor synthetic lethality with BRCA deficiency. Mol Cell.

[60] Kang Z (2021). BRCA2 associates with MCM10 to suppress PRIMPOL-mediated repriming and single-stranded gap formation after DNA damage. Nat Commun.

[61] Thakar T (2022). Lagging strand gap suppression connects BRCA-mediated fork protection to nucleosome assembly through PCNA-dependent CAF-1 recycling. Nat Commun.

[62] Vaitsiankova A (2022). PARP inhibition impedes the maturation of nascent DNA strands during DNA replication. Nat Struct Mol Biol.

[63] Tirman S (2021). Temporally distinct post-replicative repair mechanisms fill PRIMPOL-dependent ssDNA gaps in human cells. Mol Cell.

[64] Leung W (2023). ATR protects ongoing and newly assembled DNA replication forks through distinct mechanisms. Cell Rep.

[65] Schoonen PM (2017). Progression through mitosis promotes PARP inhibitor-induced cytotoxicity in homologous recombination-deficient cancer cells. Nat Commun.

[66] Noordermeer SM, van Attikum H (2019). PARP inhibitor resistance: a tug-of-war in BRCA-mutated cells. Trends Cell Biol.

[67] Sandhu D (2022). Identification of different side effects between PARP inhibitors and their polypharmacological multi-target rationale. Br J Clin Pharmacol.

[68] Knezevic CE (2016). Proteome-wide profiling of clinical PARP inhibitors reveals compound-specific secondary targets. Cell Chem Biol.

[69] Bejan DS, Cohen MS (2022). Methods for profiling the target and off-target landscape of PARP inhibitors. Curr Opin Chem Biol.

[70] Farrés J (2013). Parp-2 is required to maintain hematopoiesis following sublethal γ-irradiation in mice. Blood.

[71] Langelier M-F (2023). Clinical PARP inhibitors allosterically induce PARP2 retention on DNA. Sci Adv.

[72] Mohyuddin GR (2020). Similar response rates and survival with PARP inhibitors for patients with solid tumors harboring somatic versus Germline BRCA mutations: a Meta-analysis and systematic review. BMC Cancer.

[73] Rottenberg S, Jonkers J (2008). Modeling therapy resistance in genetically engineered mouse cancer models. Drug Resist Updat.

[74] Gogola E (2018). Selective loss of PARG restores PARylation and counteracts PARP inhibitor-mediated synthetic lethality. Cancer Cell.

[75] Bhin J (2023). Multi-omics analysis reveals distinct non-reversion mechanisms of PARPi resistance in BRCA1-versus BRCA2-deficient mammary tumors. Cell Rep.

[76] Edwards SL (2008). Resistance to therapy caused by intragenic deletion in BRCA2. Nature.

[77] Pettitt SJ (2020). Clinical *BRCA1/2* reversion analysis identifies hotspot mutations and predicted neoantigens associated with therapy resistance. Cancer Discov.

[78] Swisher EM (2017). Rucaparib in relapsed, platinum-sensitive high-grade ovarian carcinoma (ARIEL2 Part 1): an international, multicentre, open-label, phase 2 trial. Lancet Oncol.

[79] Callen E (2013). 53BP1 mediates productive and mutagenic DNA repair through distinct phosphoprotein interactions. Cell.

[80] Escribano-Díaz C (2013). A cell cycle-dependent regulatory circuit composed of 53BP1-RIF1 and BRCA1-CtIP controls DNA repair pathway choice. Mol Cell.

[81] Xu G (2015). REV7 counteracts DNA double-strand break resection and affects PARP inhibition. Nature.

[82] He YJ (2018). DYNLL1 binds to MRE11 to limit DNA end resection in BRCA1-deficient cells. Nature.

[83] Clairmont CS (2020). TRIP13 regulates DNA repair pathway choice through REV7 conformational change. Nat Cell Biol.

[84] Peng Y (2019). The deubiquitylating enzyme USP15 regulates homologous recombination repair and cancer cell response to PARP inhibitors. Nat Commun.

[85] Groelly FJ (2023). Targeting DNA damage response pathways in cancer. Nat Rev Cancer.

[86] Wang AT (2015). A dominant mutation in human RAD51 reveals its function in DNA interstrand crosslink repair independent of homologous recombination. Mol Cell.

[87] Rondinelli B (2017). EZH2 promotes degradation of stalled replication forks by recruiting MUS81 through histone H3 trimethylation. Nat Cell Biol.

[88] Hill SJ (2018). Prediction of DNA repair inhibitor response in short-term patient-derived ovarian cancer organoids. Cancer Discov.

[89] Dias MP (2021). Loss of nuclear DNA ligase III reverts PARP inhibitor resistance in BRCA1/53BP1 double-deficient cells by exposing ssDNA gaps. Mol Cell.

[90] Lim PX (2024). BRCA2 promotes genomic integrity and therapy resistance primarily through its role in homology-directed repair. bioRxiv.

[91] Zou L, Elledge SJ (2003). Sensing DNA damage through ATRIP recognition of RPA-ssDNA complexes. Science.

[92] Buisson R (2017). Coupling of homologous recombination and the checkpoint by ATR. Mol Cell.

[93] De La Cruz LM, Czerniecki BJ (2018). Immunotherapy for breast cancer is finally at the doorstep: immunotherapy in breast cancer. Ann Surg Oncol.

[94] Kim H (2017). Targeting the ATR/CHK1 axis with PARP inhibition results in tumor regression in *BRCA*-mutant ovarian cancer models. Clin Cancer Res.

[95] Kim H (2020). Combining PARP with ATR inhibition overcomes PARP inhibitor and platinum resistance in ovarian cancer models. Nat Commun.

[96] Parmar K (2019). The CHK1 inhibitor prexasertib exhibits monotherapy activity in high-grade serous ovarian cancer models and sensitizes to PARP inhibition. Clin Cancer Res.

[97] Wethington SL (2023). Combination A TR (ceralasertib) and PARP (olaparib) inhibitor (CAPRI) trial in acquired PARP inhibitor–resistant homologous recombination–deficient ovarian cancer. Clin Cancer Res.

[98] Ngoi NY (2024). Targeting ATR in patients with cancer. Nat Rev Clin Oncol.

[99] Benada J (2023). Synthetic lethal interaction between WEE1 and PKMYT1 is a target for multiple low-dose treatment of high-grade serous ovarian carcinoma. NAR Cancer.

[100] Elbæk CR (2022). WEE1 kinase protects the stability of stalled DNA replication forks by limiting CDK2 activity. Cell Rep.

[101] Serra V (2022). Identification of a molecularly-defined subset of breast and ovarian cancer models that respond to WEE1 or ATR inhibition, overcoming PARP inhibitor resistance. Clin Cancer Res.

[102] Teo ZL (2023). Combined PARP and WEE1 inhibition triggers anti-tumor immune response in BRCA1/2 wildtype triple-negative breast cancer. NPJ Breast Cancer.

[103] Karnak D (2014). Combined inhibition of Wee1 and PARP1/2 for radiosensitization in pancreatic cancer. Clin Cancer Res.

[104] Fang Y (2019). Sequential therapy with PARP and WEE1 inhibitors minimizes toxicity while maintaining efficacy. Cancer Cell.

[105] Gallo D (2022). CCNE1 amplification is synthetic lethal with PKMYT1 kinase inhibition. Nature.

[106] Ceccaldi R (2015). Homologous-recombination-deficient tumours are dependent on Polθ-mediated repair. Nature.

[107] Belan O (2022). POLQ seals post-replicative ssDNA gaps to maintain genome stability in BRCA-deficient cancer cells. Mol Cell.

[108] Zhou J (2021). A first-in-class polymerase theta inhibitor selectively targets homologous-recombination-deficient tumors. Nat Cancer.

[109] Zatreanu D (2021). Polθ inhibitors elicit BRCA-gene synthetic lethality and target PARP inhibitor resistance. Nat Commun.

[110] Lim KS (2018). USP1 is required for replication fork protection in BRCA1-deficient tumors. Mol Cell.

[111] Da Costa AABA (2023). The USP1 inhibitor I-138 kills BRCA1-deficient tumor cells and overcomes PARP inhibitor resistance. Cancer Res.

[112] Simoneau A (2023). Characterization of the clinical development candidate TNG348 as a potent and selective inhibitor of USP1 for the treatment of BRCA1/2mut cancers. Cancer Res.

[113] Fugger K (2021). Targeting the nucleotide salvage factor DNPH1 sensitizes *BRCA*-deficient cells to PARP inhibitors. Science.

[114] Bonagas N (2022). Pharmacological targeting of MTHFD2 suppresses acute myeloid leukemia by inducing thymidine depletion and replication stress. Nat Cancer.

[115] Verma P (2021). ALC1 links chromatin accessibility to PARP inhibitor response in homologous recombination-deficient cells. Nat Cell Biol.

[116] Blessing C (2020). The oncogenic helicase ALC1 regulates PARP inhibitor potency by trapping PARP2 at DNA breaks. Mol Cell.

[117] Hewitt G (2021). Defective ALC1 nucleosome remodeling confers PARPi sensitization and synthetic lethality with HRD. Mol Cell.

[118] Schleicher EM, Moldovan GL (2022). CRISPR screens guide the way for PARP and ATR inhibitor biomarker discovery. FEBS J.

[119] Zimmermann M (2018). CRISPR screens identify genomic ribonucleotides as a source of PARP-trapping lesions. Nature.

[120] Tsujino T (2023). CRISPR screens reveal genetic determinants of PARP inhibitor sensitivity and resistance in prostate cancer. Nat Commun.

[121] Ipsen MB (2022). A genome-wide CRISPR-Cas9 knockout screen identifies novel PARP inhibitor resistance genes in prostate cancer. Oncogene.

[122] Mendes-Pereira AM (2009). Synthetic lethal targeting of PTEN mutant cells with PARP inhibitors. EMBO Mol Med.

[123] Sulkowski PL (2017). 2-Hydroxyglutarate produced by neomorphic IDH mutations suppresses homologous recombination and induces PARP inhibitor sensitivity. Sci Transl Med.

[124] Schvartzman JM (2023). Oncogenic IDH mutations increase heterochromatin-related replication stress without impacting homologous recombination. Molecular Cell.

[125] Brenner JC (2012). PARP-1 inhibition as a targeted strategy to treat Ewing’s sarcoma. Cancer Res.

[126] Gorthi A (2018). EWS-FLI1 increases transcription to cause R-loops and block BRCA1 repair in Ewing sarcoma. Nature.

[127] Li L (2017). Androgen receptor inhibitor-induced “BRCAness” and PARP inhibition are synthetically lethal for castration-resistant prostate cancer. Sci Signal.

[128] Asim M (2017). Synthetic lethality between androgen receptor signalling and the PARP pathway in prostate cancer. Nat Commun.

[129] Kawale AS (2024). APOBEC3A induces DNA gaps through PRIMPOL and confers gap-associated therapeutic vulnerability. Sci Adv.

[130] McCabe N (2006). Deficiency in the repair of DNA damage by homologous recombination and sensitivity to poly(ADP-ribose) polymerase inhibition. Cancer Res.

[131] Lord CJ (2008). A high-throughput RNA interference screen for DNA repair determinants of PARP inhibitor sensitivity. DNA Repair (Amst).

[132] Pantelidou C (2019). PARP inhibitor efficacy depends on CD8^+^ T-cell recruitment via intratumoral STING pathway activation in BRCA-deficient models of triple-negative breast cancer. Cancer Discov.

[133] Ding L (2018). PARP Inhibition elicits STING-dependent antitumor immunity in Brca1-deficient ovarian cancer. Cell Rep.

[134] Ding L (2023). STING agonism overcomes STAT3-mediated immunosuppression and adaptive resistance to PARP inhibition in ovarian cancer. J Immunother Cancer.

[135] Murai J, Pommier Y (2019). PARP trapping beyond homologous recombination and platinum sensitivity in cancers. Ann Rev Cancer Biol.

[136] Wahlberg E (2012). Family-wide chemical profiling and structural analysis of PARP and tankyrase inhibitors. Nat Biotechnol.

[137] Dias MP (2021). Understanding and overcoming resistance to PARP inhibitors in cancer therapy. Nat Rev Clin Oncol.

[138] Wang H (2020). Discovery of Pamiparib (BGB-290), a potent and selective poly (ADP-ribose) polymerase (PARP) inhibitor in clinical development. J Med Chem.

